# pHLIP-mediated targeting of truncated tissue factor to tumor vessels causes vascular occlusion and impairs tumor growth

**DOI:** 10.18632/oncotarget.4395

**Published:** 2015-06-25

**Authors:** Suping Li, Yanhua Tian, Ying Zhao, Yinlong Zhang, Shishuai Su, Jing Wang, Meiyu Wu, Quanwei Shi, Gregory J. Anderson, Johannes Thomsen, Ruifang Zhao, Tianjiao Ji, Jie Wang, Guangjun Nie

**Affiliations:** ^1^ CAS Key Laboratory for Biomedical Effects of Nanomaterials & Nanosafety, National Center for Nanoscience and Technology, China, Beijing 100190, China; ^2^ Peking-Tsinghua Center for Life Sciences, Academy for Advanced Interdisciplinary Studies, Peking University, Beijing 100871, China; ^3^ QIMR Berghofer Medical Research Institute, Brisbane QLD 4006, Australia; ^4^ Sino-Danish Center for Education and Research, University of Chinese Academy of Sciences, Beijing 100190, China; ^5^ Department of Thoracic Medical Oncology, Peking University Cancer Hospital and Institute, Beijing 100142, China

**Keywords:** pH low insertion peptide (pHLIP), truncated tissue factor (tTF), tumor vessel targeting, thrombosis

## Abstract

Occluding tumor blood supply by delivering the extracellular domain of coagulation-inducing protein tissue factor (truncated tissue factor, tTF) to tumor vasculature has enormous potential to eliminate solid tumors. Yet few of the delivery technologies are moved into clinical practice due to their non-specific tissue biodistribution and rapid clearance by the reticuloendothelial system. Here we introduced a novel tTF delivery method by generating a fusion protein (tTF-pHLIP) consisting of tTF fused with a peptide with a low pH-induced transmembrane structure (pHLIP). This protein targets the acidic tumor vascular endothelium and effectively induces local blood coagulation. tTF-pHLIP, wherein pHLIP is cleverly designed to mimic the natural tissue factor transmembrane domain, triggered thrombogenic activity of the tTF by locating it to the endothelial cell surface, as demonstrated by coagulation assays and confocal microscopy. Systemic administration of tTF-pHLIP into tumor-bearing mice selectively induced thrombotic occlusion of tumor vessels, reducing tumor perfusion and impairing tumor growth without overt side effects. Our work introduces a promising strategy for using tTF as an anti-cancer drug, which has great potential value for clinical applications.

## INTRODUCTION

One major obstacle to the development of coagulation therapy for cancer is the inability to target vascular occluding agents specifically to tumor blood vessels, thereby limiting their therapeutic effect and causing adverse side effects. Over the past two decades, approaches to targeted coagulation therapy *in vivo* have largely focused on selective delivery of the extracellular domain of the coagulation-inducing protein tissue factor (truncated tissue factor, tTF, the initiator of the extrinsic pathway of blood coagulation) to tumor vessels, by using antibody or peptide ligands that recognize various tumor endothelial markers [1–7]. Free tTF is soluble and inactive [8–11]; however, its potent coagulation activity is recovered when localized near a phospholipid membrane. Although a number of tTF delivery strategies have been proven to selectively induce thrombotic occlusion of tumor vessels and subsequent tumor necrosis, most have not been moved into clinical practice due to nonspecific delivery and rapid clearance by the reticuloendothelial system [1, 12, 13].

The pH (low) membrane insertion peptide (pHLIP) can insert into cell membranes by forming an inducible transmembrane α-helix under acidic conditions [14–20], and, when administered systemically, is capable of targeting a variety of solid tumors because of acidosis of tumor tissues [21–25], while avoiding the liver. Based on the well-characterized tumor-targeting property of the pHLIP, we constructed a tumor-targeted tTF delivery vector (tTF-pHLIP) by fusing pHLIP to tTF. Unlike other current delivery strategies wherein tTF was delivered by targeted ligands [1–6], pHLIP-directed membrane insertion under acidic conditions could allow tTF to adopt a state which is close to the native extracellular domain of TF on membrane surfaces, thus maintaining its maximum coagulation activity.

**Figure 1:**
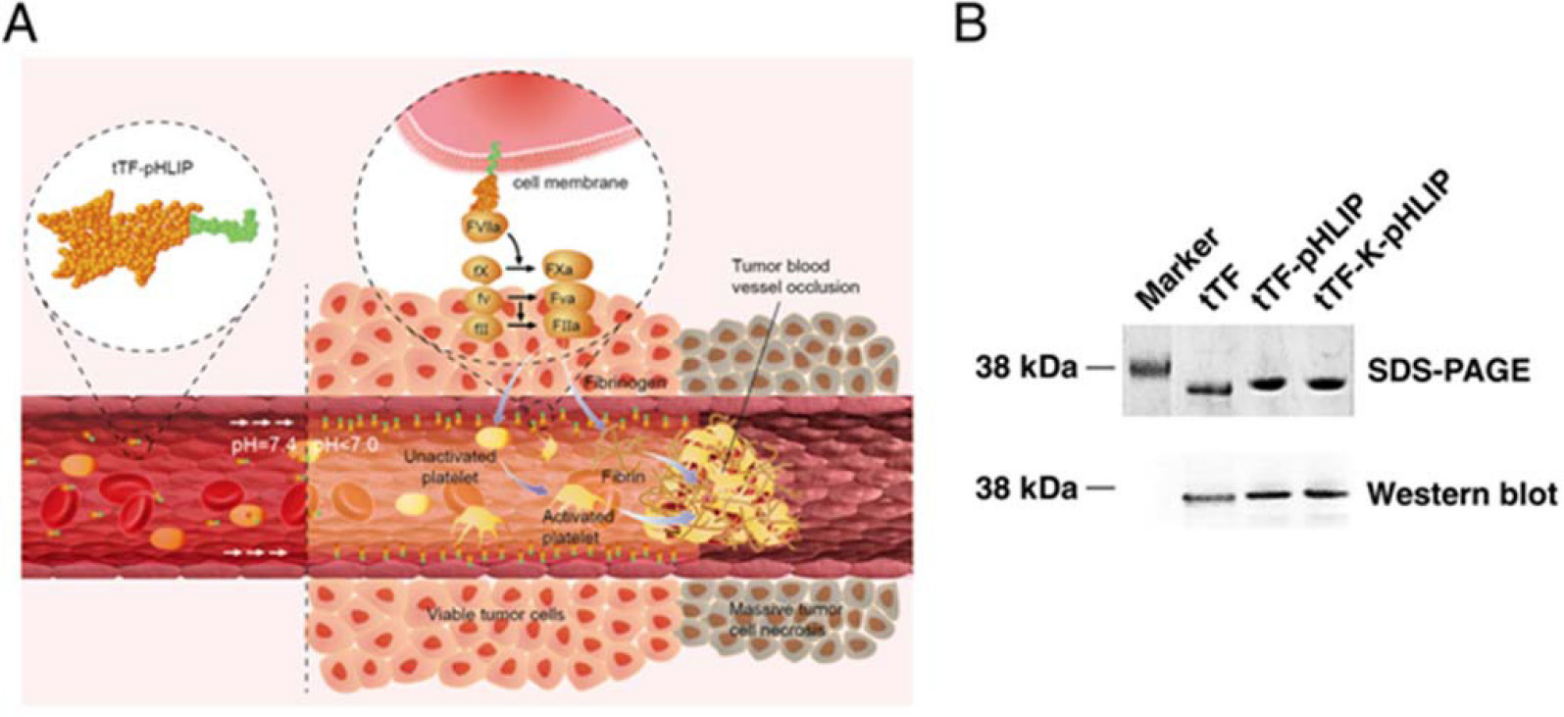
Proposed mechanism of action and characterization of fusion proteins **A.** Schematic showing the proposed mechanism of action of tTF-pHLIP within tumor blood vessels. tTF-pHLIP freely circulates in the blood at physiological pH, but inserts across the plasma membrane of tumor endothelial cells by virtue of an α-helix (green) which forms at reduced pH in tumor blood vessels. Membrane-bound tTF then triggers the blood coagulation cascade, resulting in thrombosis and consequently tumor vessel infarction and tumor cell necrosis. **B.** SDS-PAGE and western blot analysis of recombinant purified tTF-pHLIP and control proteins tTF and tTF-K-pHLIP.

We have generated the tTF-pHLIP fusion protein in which the N-terminus of pHLIP was fused to the C-terminal region of tTF, and found that tTF-pHLIP can localize to the acidic tumor endothelium of blood vessels in tumor-bearing mice and induce local intravascular thrombosis (Figure 1A), resulting in tumor infarction and regression without overt side effects.

## RESULTS

### Generation and characterization of tTF-pHLIP and control proteins

The chimeric protein tTF-pHLIP was generated by fusing the N-terminus of pHLIP to the C-terminus of the extracellular domain of tissue factor (1–218 amino acids). We also generated free tTF and tTF-K-pHLIP as controls. K-pHLIP is a mutant of pHLIP where the residues Asp 13 and Asp 24 in the transmembrane segment of pHLIP are replaced by Lys residues. This leads to the loss of the pH-dependent membrane insertion function [26, 27]. SDS-PAGE analysis of the purified proteins showed the expected sizes, with M_r_ values of approximate 32, 000 Da for tTF-pHLIP, 28, 000 Da for tTF and 32, 000 Da for tTF-K-pHLIP (Figure 1B). Western blotting using monoclonal anti-human tissue factor antibody further confirmed the presence of the tTF moiety in these three proteins (Figure 1B).

### Functional characterization of tTF-pHLIP fusion proteins

**Figure 2:**
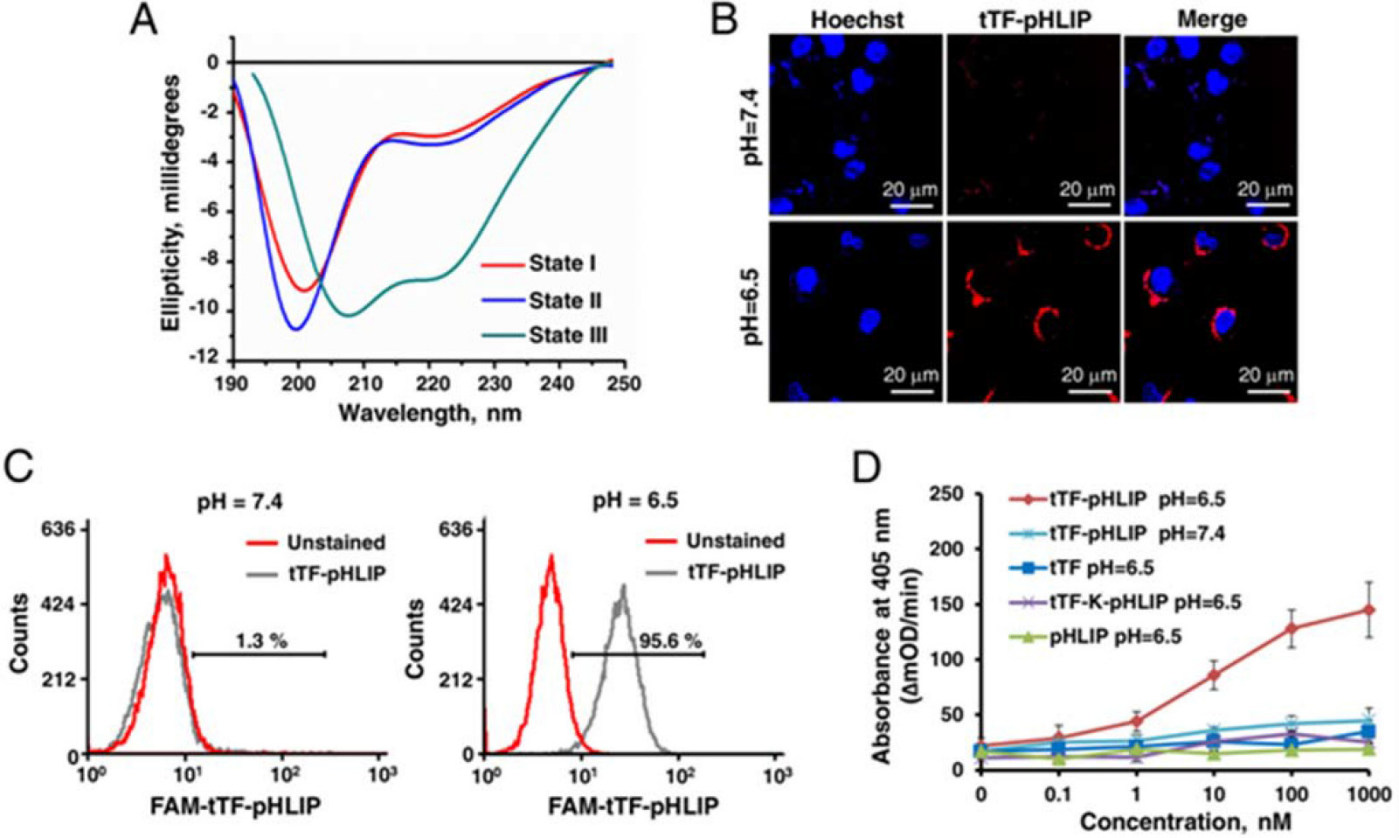
Functional characterization of expressed tTF-pHLIP proteins *in vitro* **A.** Circular dichroism (CD) spectral signals of tTF-pHLIP. The CD spectra of tTF-pHLIP at pH7.4 in the absence (State I) or presence of liposomes (state II) indicated an unstructured configuration of pHLIP peptide. Decreasing the pH to 6.5 (State III) induced the membrane insertion of pHLIP via α-helix formation. **B.** Confocal images of HUVECs incubated with Alexa 594-labeled tTF-pHLIP (1.24 μM, red) at pH 7.4 (top) or 6.5 (bottom) for 30 min. Cell nuclei were labeled with Hoechst 33342 (blue). **C.** HUVECs were incubated in PBS at pH 7.4 or 6.5 in the presence (gray) or absence (red) of Alexa 594-labeled tTF-pHLIP (40 μg) at 37°C for 1 hour. Cell surface binding of Alexa 594-labeled tTF-pHLIP was assessed by flow cytometry. **D.** The ability of tTF-pHLIP and control proteins to facilitate the specific proteolytic activation of factor X by factor VIIa in the presence of HUVECs at pH 7.4 or 6.5 was assessed using a Spectozyme FXa assay. pHLIP was used as negative control. Data represent the mean ± SD of five independent experiments.

We first tested whether tTF-pHLIP has the ability to insert into the lipid bilayer by using circular dichroism (CD) spectra. The CD spectral signal has been used to monitor the conformational changes of pHLIP in a free state at neutral pH to α-helix formation when inserted into bilayers at lower pH [17]. Our data showed that pHLIP fused with tTF was predominantly unstructured at pH 7.4 in the absence or presence of POPC liposomes (Figure 2A and Supplementary Figure S1 for liposome characteristics) [28, 29], whereas α-helix formation was observed at pH 6.5 in the presence of liposomes, as identified by the characteristic negative peaks at 208 nm and 222 nm. Thus, we conclude that the conjugation of tTF to the N-terminus of pHLIP did not affect the ability of the peptide to form a helical structure and to insert into the membrane bilayers at low pH.

Next, we evaluated the interaction of tTF-pHLIP with the cell membrane at neutral and acidic pH values by confocal microscopy and flow cytometry. In the confocal microscopy assays, HUVEC cells were incubated with Alexa 594-labeled tTF-pHLIP (1.24 μM) for 30 min at 37°C, at either pH 7.4 or 6.5. Our results demonstrated that bare fluorescence was found on the tTF-pHLIP-treated cell membrane at pH 7.4 (Figure 2B). In contrast, strong fluorescent signals were observed on the cell surface at pH 6.5 (Figure 2B), revealing the membrane insertion of the pHLIP at low pH. Specific binding of tTF-pHLIP to the cells at low pH was also determined by flow cytometry (Figure 2C). To determine whether tTF-pHLIP bound to cells could induce blood coagulation, we evaluated the ability of tTF-pHLIP to enhance the specific proteolytic activity of factor X by factor VIIa, a key step in the extrinsic coagulation pathway [8–11]. HUVECs were incubated with tTF-pHLIP in the presence of factor VIIa, followed by the addition of substrate factor X. At pH 7.4, tTF-pHLIP was unable to induce factor X activation (Figure 2D); however, at pH 6.5, tTF-pHLIP was found to activate factor X in a concentration-dependent manner. Activation of factor X at lower pH was dependent on the presence of pHLIP, as free tTF or the mutant tTF-K-pHLIP could not induce it. Together, these data indicate that the ligation of the pHLIP peptide to the C-terminus of tTF could target tTF to the cell membrane while not influencing its coagulation activity.

### *In vivo* tumor vessel targeting and intravascular thrombosis

**Figure 3:**
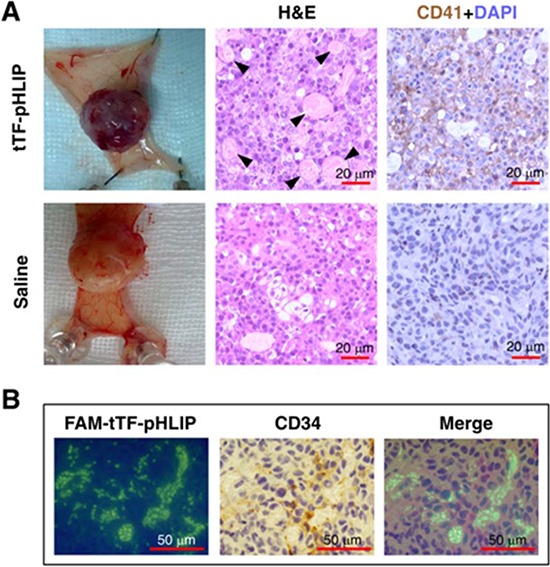
tTF-pHLIP treatment induces thrombosis within tumor vessels, and tumor vessel accumulation of tTF-pHLIP **A.** MDA-MB-231 human breast cancer-bearing nude mice were injected with tTF-pHLIP or saline via a tail vein and the tumors were resected 8 hours post-injection. The tumors of mice treated with tTF-pHLIP were bruised and blackened (top left) in contrast to the vital appearance of the tumors in mice treated with saline (bottom left), indicating blood pooling due to vascular disruption. Tumor sections were subjected to hematoxylin and eosin (H&E) staining to detect thrombosis (middle, arrow). Immunostaining of tumor sections with anti-mouse platelet CD41 antibody further confirmed thrombosis in the tumors of tTF-pHLIP-treated mice (top right, darker brown). Data are representative of at least three separate experiments. **B.** FAM-labeled tTF-pHLIP was injected intravenously into nude mice bearing MDA-MB-231 tumors. Tumors were harvested 8 hours later, and tumor sections were stained with anti-CD34 antibody and examined by confocal microscopy. The tTF-pHLIP was green; blood vessels were visualized with anti-CD34 (brown); nuclei were stained with DAPI (blue).

**Figure 4:**
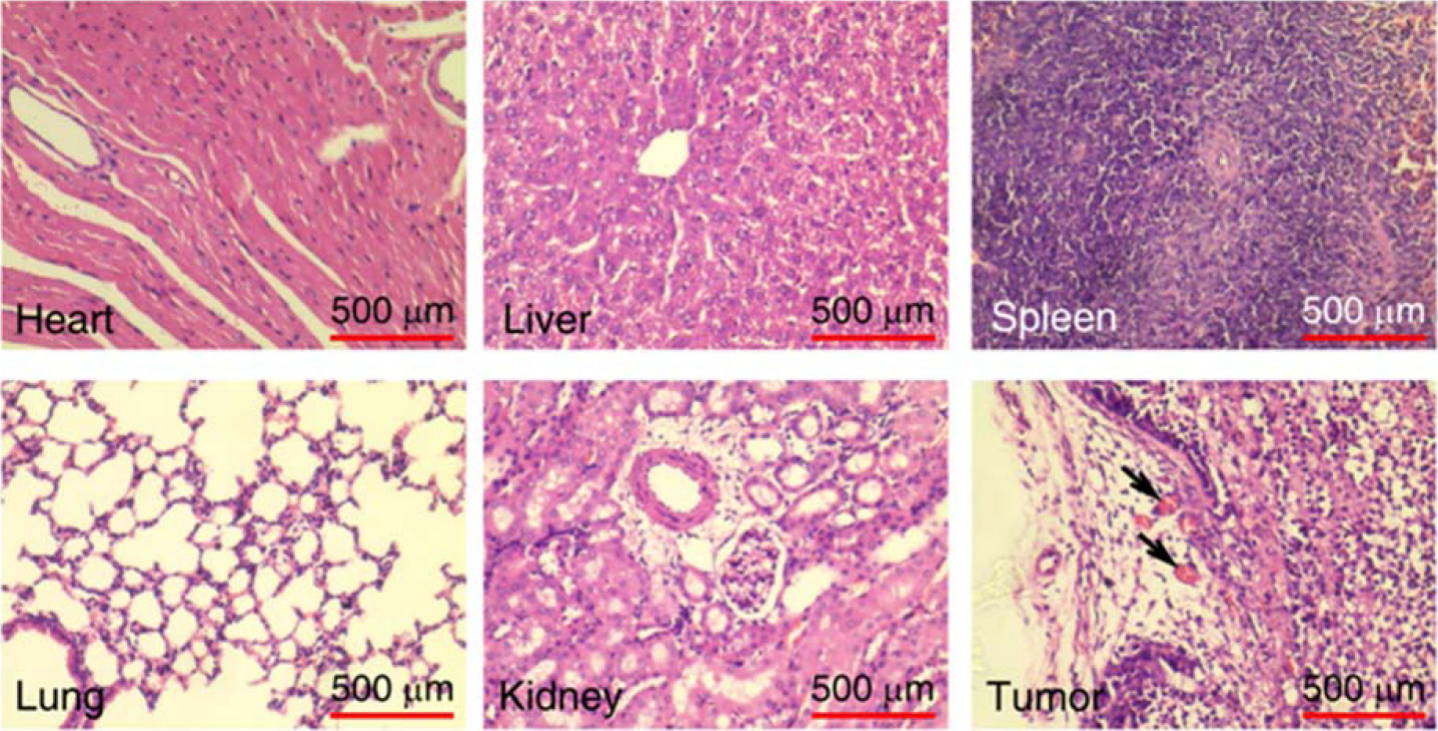
Thrombotic risk assessment in the normal tissue of the tTF-pHLIP-treated mice Histological analysis of various normal organs and tumor of MDA-MB-231 tumor-bearing mice 24 hours after treatment with tTF-pHLIP. Sections of heart, liver, spleen, lung, kidney and tumor were stained with hematoxylin and eosin (H&E). Thrombosis was only found in the blood vessels of the tumor, as indicated by the arrows.

We next tested the tumor vascular targeting capacity of tTF-pHLIP in nude mice bearing established ∼100 mm^3^ orthotopic MDA-MB-231 human breast xenograft tumors. A 20 μg/mouse (equivalent to approximately 833 μg/kg) dose of tTF-pHLIP was injected into the mice via the tail vein, with saline as a negative control. Note that this dose was sufficiently safe as demonstrated in a dose escalation study (Supplementary Figures S2-S3 and Supplementary Videos 1–3). After 8 hours we removed the tumors and found apparent hemorrhage in tumors treated with tTF-pHLIP (Figure 3A, top left) compared with the vital appearance of saline-treated tumors (Figure 3A, bottom left), indicating blood pooling due to vascular disruption. Hematoxylin and eosin (H&E) accordingly revealed extensive thrombosis in the blood vessels of tumors treated with tTF-pHLIP but not saline (Figure 3A, middle). We also stained the tumors with a CD41-specific antibody to further identify thrombosis, as demonstrated by the presence of activated platelet aggregates (Figure 3A, right). Biodistribution data revealed that tTF-pHLIP was specifically accumulated in the tumor 8 hours after intravenous administration, but little or no accumulation in heart, liver, spleen, kidney or lung (Supplementary Figure S4), suggesting a high specificity of tTF-pHLIP for tumor tissue. Accordingly, H&E staining showed no thrombosis in the blood vessels of all these normal tissues of the mice for up to 24 hours after tTF-pHLIP treatment (Figure 4).

We further confirmed whether tTF-pHLIP accumulated in the vessels of tumors and thus caused clotting in them. For tumors of mice treated with FAM (5(6)-carboxyfluorescein)-labeled tTF-pHLIP, staining of tumor sections with antibody against vascular endothelial cell marker CD34 showed the binding of tTF-pHLIP to the blood vessel walls as verified by colocalization with CD34 (Figure 3B).

### tTF-pHLIP reduces tumor perfusion and promotes tumor regression

**Figure 5:**
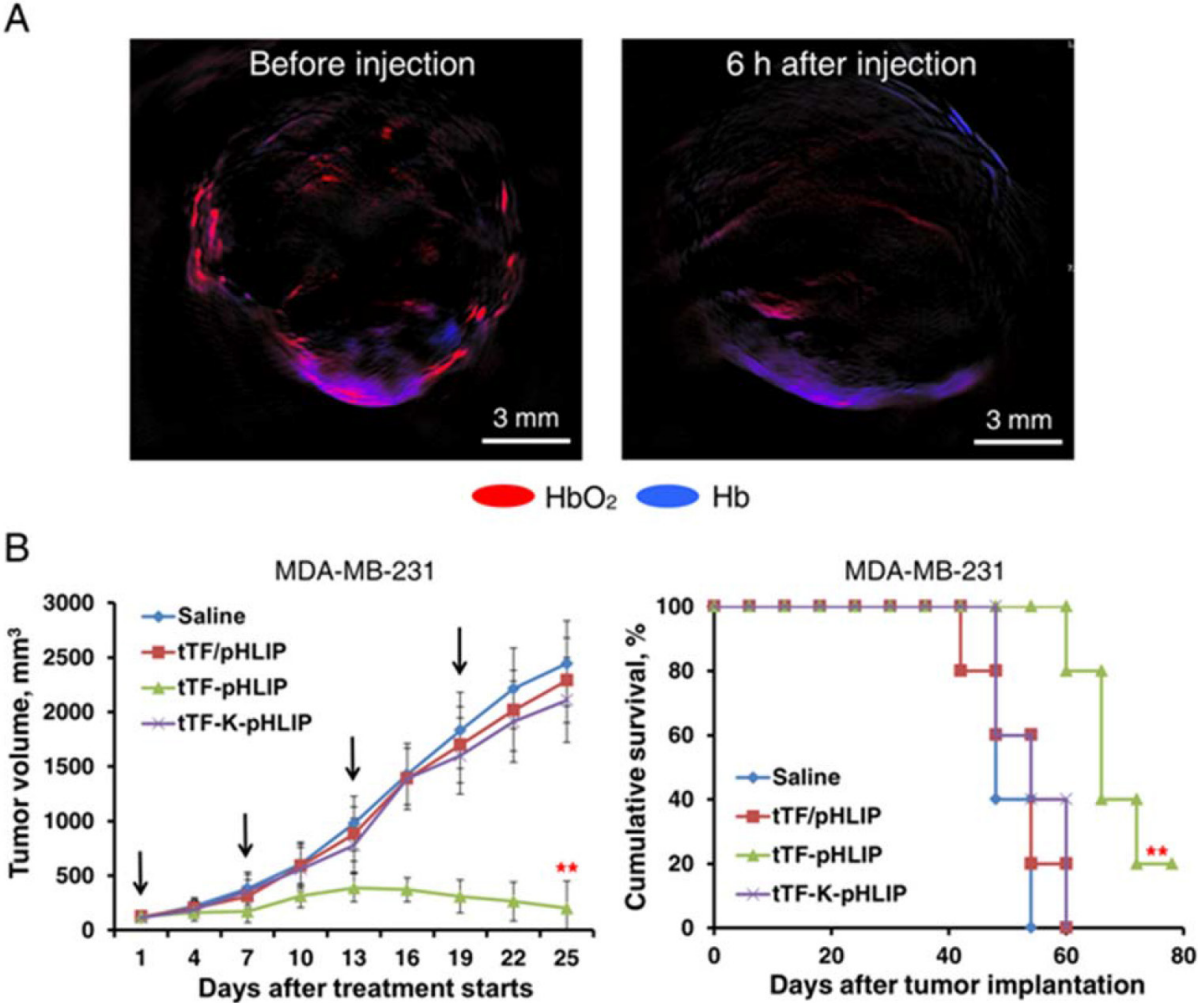
Photoacoustic (PA) imaging of blood supply in tumors of mice treated with tTF-pHLIP and antitumor activity of tTF-pHLIP in model of MDA-MB-231 cancer **A.** MDA-MB-231 tumor-bearing nude mice were injected intravenously with tTF-pHLIP. After 6 hours, PA signals of oxyhemoglobin (HbO_2_, red) and hemoglobin (Hb, blue) within the tumors were collected to detect the change of blood supply. tTF-pHLIP treatment leads to a significant reduction in tumor perfusion. The images are representative of 3 tumors imaged. **B.** Nude mice bearing ∼100 mm^3^ MDA-MB-231 tumors were injected intravenously with various vehicles at the indicated time (arrows), and tumor sizes were recorded thereafter. Each bar represents the mean ± SD. **P < 0.01 by ANOVA for tumor volume (left) and log-rank [Mantel-Cox] test for survival analysis (right); *n* = 6 – 7 mice per group.

To assess the impact of tTF-pHLIP-induced thrombosis on blood supply, we analyzed tumor blood circulation in MDA-MB-231 tumor-bearing nude mice using photoacoustic imaging [30]. This technique evaluates blood perfusion of the target organ by comparing the characteristic signals of oxyhemoglobin (HbO_2_) and hemoglobin (Hb). Our results showed that in contrast to the pre-injection state, the HbO_2_ signal declined sharply while the Hb signal increased considerably 6 hours after tTF-pHLIP treatment (Figure 5A), demonstrating a decrease in blood supply due to thrombotic occlusion.

We further tested the therapeutic efficacy of the tTF-pHLIP treatment. Nude mice bearing established ∼100 mm^3^ MDA-MB-231 tumors were randomly sorted into four groups and were treated with saline, tTF-K-pHLIP, tTF in combination with free pHLIP (tTF/pHLIP) or tTF-pHLIP by the tail vein 4 times at intervals of 6 days. After the second injection, a reduction in tumor volume was observed in mice treated with tTF-pHLIP compared with the saline group, and this became statistically significant from day 13 onwards. In contrast, the tumors continued to grow in all other treatment groups, and none of these groups were significantly different from the saline control. After the fourth injection, tTF-pHLIP-treated mice had a mean tumor volume of 552 vs 2443 mm^3^ for saline, 2109 mm^3^ for tTF-K-pHLIP and 2289 mm^3^ for the tTF/pHLIP group (Figure 5B, left). The effective inhibition of tumor growth by tTF-pHLIP correlated with a substantial increase in median survival of animals (69.2 days vs 48.4 for saline, 49.6 for tTF-K-pHLIP and 49.0 days for tTF/pHLIP group) (Figure 5B, right).

**Figure 6:**
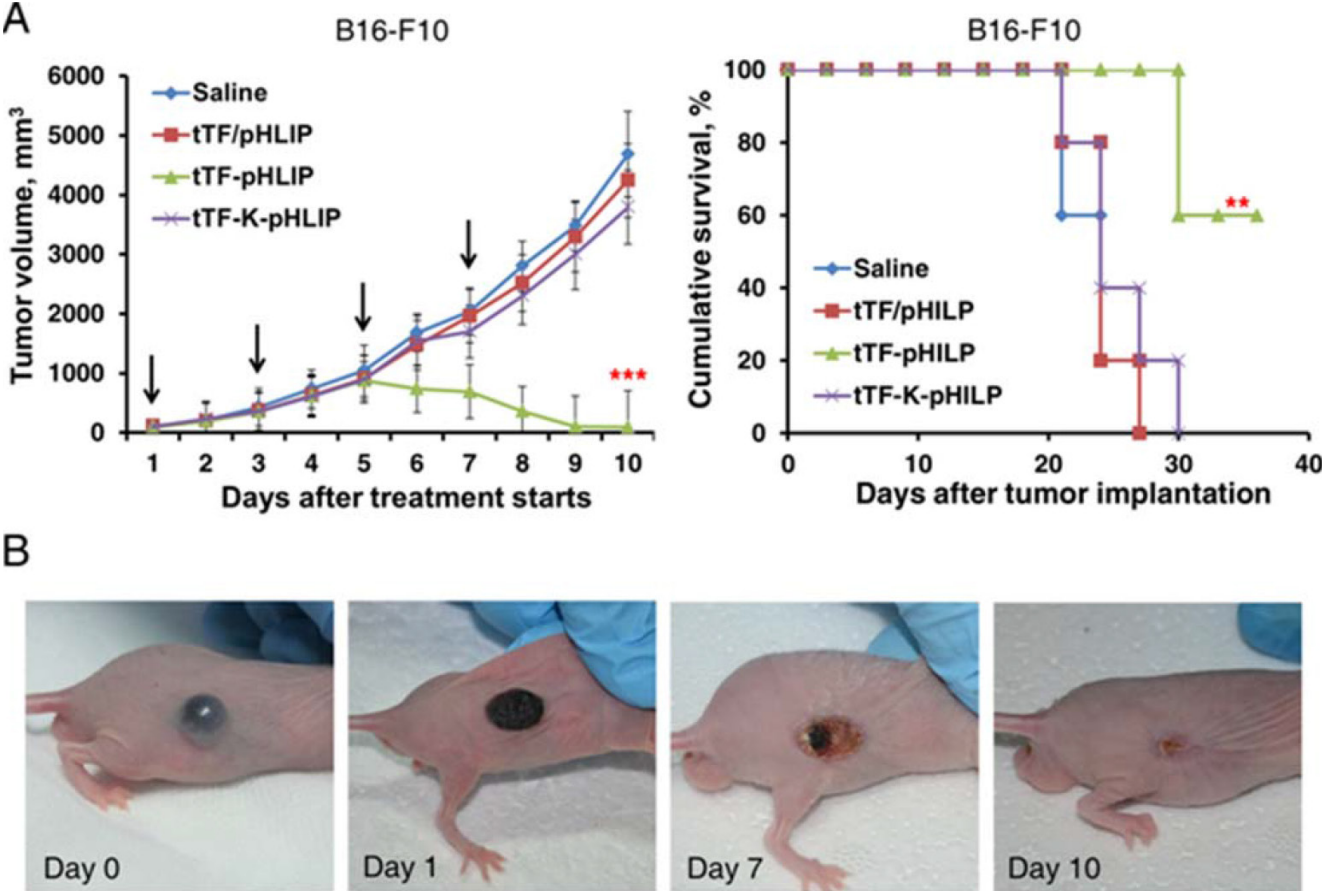
Antitumor activity of tTF-pHLIP in B16-F10 melanoma model **A.** C57BL/6 mice bearing ∼150 mm^3^ melanomas were injected with various vehicles every other day by the tail vein, and tumor sizes were recorded thereafter. Each bar represents the mean ± SD. **P < 0.01 and ***P < 0.001 by ANOVA for tumor volume (left) and log-rank [Mantel-Cox] test for survival analysis (right); *n* = 6 - 7 mice per group. **B.** Gross appearance of B16-F10 tumors in BALB/c nude mice after treatment with tTF-pHLIP. At the beginning of treatment, the tumor was well-developed and growing rapidly. One day after treatment, the tumor began to scab. The scabs came off with time, and by day 10 only fibrous scar tissue was visible in four of the six mice.

The antitumor activity of tTF-pHLIP was further determined in C57BL/6 or BALB/c nude mice bearing established ∼150 mm^3^ B16-F10 tumors. For C57BL/6 mice, tTF-pHLIP or various controls were administered intravenously 4 times at intervals of 48 hours. Mice injected with saline, tTF-K-pHLIP or tTF/pHLIP formed large tumors (Figure 6A, left). In contrast, mice injected with tTF-pHLIP displayed rapid tumor regression. The median survival time for saline-treated mice was 22.8 days (Figure 6A, right), and treatment with tTF-K-pHLIP or tTF/pHLIP did not increase this survival. However, the tTF-pHLIP allowed survival of four of six mice for up to 43.4 days or more after tumor implantation. This curative effect was also demonstrated in the B16-F10 nude mouse model (Figure 6B).

### Safety and tolerability

During the course of the therapeutic experiments, no morbidity or mortality was observed in both types of tumor-bearing mice after a total of 4 intravenous injections of tTF-pHLIP (20 μg/mouse for each injection) at the indicated time intervals as mentioned above. This indicates that tTF-pHLIP was not overtly toxic to the animals. Histological analysis also showed no visible thrombosis in the major organs (heart, liver, spleen, lung and kidney) of the MDA-MB-231 tumor-bearing mice after single or multiple (3 times) tTF-pHLIP administrations (Supplementary Figure S5), indicating that the tTF-pHLIPs are well-tolerated.

To investigate whether tTF-pHLIP induces an innate immune response, we assessed IL-6, IP-10, TNF-α and IFN-α serum concentrations of non-tumor-bearing C57BL/6 mice before and after tTF-pHLIP treatment. No significant changes in concentration of any of the cytokines were found after single or multiple (3 times) injections of tTF-pHLIP (Supplementary Table S1), revealing that tTF-pHLIP may be immunologically inert.

## DISCUSSION

A number of methods for tTF delivery have previously been described by fusing tTF to peptide or antibody ligands that target various overexpressed tumor vascular markers [1–6]. However, most of these ligands recognize markers that are also constitutively expressed in normal endothelial cells, thereby causing off-target effects. Another major problem is steric effects related to ligand-receptor binding, wherein a bound receptor covers neighboring ligand site due to its physical size, leading to steric exclusion of ligand sites near a bound receptor and thus reducing the average reactivity of neighboring ligand sites [31].

In contrast, we have found in this study that pHLIP technology can be used to selectively deliver tTF to tumor blood vessels based on the ability of pHLIP to undergo membrane insertion in acidic tumor vascular compartments [32]. pHLIP can exist in three states: in a water soluble form, bound to a membrane surface and inserted across the lipid membrane as an α-helix [14–17]. At physiological pH, pHLIP is largely water-soluble, whereas at slightly acidic pH, pHLIP forms an α-helix which is capable of inserting into a lipid bilayer. This characteristic makes pHLIP an attractive targeting moiety for selectively labeling and tracing acidic tissues *in vivo* [14]. We generated the fusion protein tTF-pHLIP in which the pHLIP peptide is able to form α-helix to insert across tumor endothelium in response to acidic tumoral pH, thereby locating tTF on plasma membrane surface. Therefore, a distinctive feature of pHLIP-based targeting of tTF is that pHLIP binding does not require ligand-receptor interaction as used in previous tTF targeting strategies [1–6], but depends on a drop in tumor vascular pH. More importantly, in contrast to the existing tTF delivery methods, the pHLIP peptide in tTF-pHLIP functions similarly to the transmembrane domain of native TF protein, and this should confer tTF a state similar to the native extracellular domain of TF. As a result, the blood coagulation function of tTF can be better recovered in tTF-pHLIP compared to ligand-mediated targeting.

We have demonstrated that systemic administration of tTF-pHLIP can specifically induce thrombotic occlusion of tumor vessels and impairs tumor growth in two mouse tumor models: MDA-MB-231 human breast orthotopic xenograft tumor and B16-F10 melanoma. Thrombosis or other abnormalities were not observed in normal organs of the mice. The selective binding of tTF-pHLIP fusion protein to tumor endothelium is in accordance with the prior findings of pHLIP targeting of acidic tumors [21–25]. Furthermore, although tTF-pHLIP *in vivo* demonstrated no overt immune stimulation, its possible impact on the endotoxin levels will need to be further characterized.

In conclusion, we have shown that the fusion protein tTF-pHLIP is an effective antitumor molecule by targeting tumor vessels and inducing vascular infarction. This study, we believe, represents the first time pHLIP technology has been introduced into cancer therapy and this new anticancer treatment approach is uniquely attractive for moving into clinical practice.

## MATERIALS AND METHODS

### Cloning, expression and purification of tTF, tTF-K-pHLIP and tTF-pHLIP

The cDNAs coding for tTF (amino acids 1 to 218 of human TF) and tTF-pHLIP in which the pHLIP polypeptide (cDNA sequence: GCTGAACAGAACCCGATCTACTGGGCTCGTTACG CTGACTGGCTGTTCACCACCCCGCTGCTGCTGCT GGACCTGGCTCTGCTGGTTGACGCTGACGAAGG TACC, amino acid sequence: AEQNPIYWARYADWLFTTPLLLLDLALLVDADEGT) was linked to the C-terminus of tTF, were synthesized *de novo.* The cDNAs were cloned into *Nde* I and *Xho* I sites of the expression vector pET-30a(+). The mutant control tTF-K-pHLIP was produced by replacing pHLIP in the tTF-pHLIP with K-pHILP (changes underlined) (cDNA sequence: GCTGAACAGAACCCGATCTACTGGGCTCGTTACG CTAAATGGCTGTTCACCACCCCGCTGCTGCTGCTG AAACTGGCTCTGCTGGTTGACGCTGACGAAGGT ACC; amino acid sequence: AEQNPIYWARYAKW LFTTPLLLLKLALLVDADEGT) using the same approach. A cDNA encoding tTF containing a hexahistidine-tag at the 5′-end was amplified by polymerase chain reaction (PCR) using the primer pair 5′- ATTATATGCTGCCACAGTATTTGTAGTGCCTGA CATATGCC-3′ (5′-primer) and 5′- GGATTCAGAGA AGAGCACCACCACCACCACCACTGAGAGCTCC-3′ (3′-primer), and subcloned into the expression vector pET-30(+)a at the *Nde* I and *Xho* I sites. The vectors were introduced into competent *E. coli* cells (BL21DE3) according to the manufacturer's protocol (Invitrogen, Carlsbad, CA). After stimulating with IPTG (0.5 mM) for 4 hours, the cells were harvested and homogenized by sonication in washing buffer (25 mL 10 mM Tris-HCl, pH 8.0). To solubilize inclusion bodies, 5 to 10 mL of urea buffer (10 mM Tris-HCl, pH 8.0, containing 8 M urea) was added and the samples were incubated overnight at room temperature (RT). The suspension was centrifuged at 5,000 × *g* for 10 min at 4°C. The supernatant was filtered through a 0.22 μm filter and loaded onto a nickel-nitrilotriacetic acid column (Ni-NTA, Novagen, Carlsbad, CA). Purification and refolding were carried out using a His Bind Buffer Kit (BD Pharmingen, San Diego, CA) according to the manufacturer's instructions. To remove the salt, the protein solution was dialyzed in a Slide-A-Lyzer^R^10 K dialysis cassette (Pierce, Bonn, Germany) against Tris-buffered saline (TBS) (20 mM Tris, 150 mM NaCl, pH7.4). Subsequently, tTF and tTF-pHILP were analyzed under denaturing conditions by sodium dodecyl sulfate polyacrylamide gel electrophoresis (SDS-PAGE) and western blotting using mouse monoclonal anti-human tissue factor antibody (R&D Systems, 1:1000).

### Factor X activation by tTF, tTF-K-pHLIP and tTF-pHLIP

The ability of tTF, tTF-K-pHLIP and tTF-pHLIP to facilitate the specific proteolytic activation of factor X by factor VIIa was assessed as described by Ruf *et al.* [33]. In brief, POPC liposomes were mixed with factor VIIa (10 nM) (BD Pharmingen, San Diego, CA) in a total volume of 100 μL. Recombinant proteins were added in a volume of 10 μL to final concentrations ranging from 1 to 50 nM. After 5 min at 37°C, 50 μL of factor X was added to a final concentration of 30 nM for an additional 5 min at RT. Spectrozyme FXa (100 μL) (BD Pharmingen, San Diego, CA) was then added to reach a final concentration of 0.3 mM, and absorption at 405 nm (absorption maximum for the product generated by proteolytic cleavage of the substrate by Xa) was measured after 3 min. In cell-based assays, human umbilical vein endothelial cells (HUVECs, American Type Culture Collection) were premixed with factor VIIa (10 nM) in a total volume of 100 μL, and factor X activation was analyzed.

### Circular dichroism

Circular dichroism (CD) measurements were carried out on a Bio-Logic Modular Optical System 450 (MOS-450) at 25°C in phosphate buffer. Briefly, POPC liposomes (700 μM) and tTF-pHLIP (10 μM) were mixed in 10 mM phosphate buffer. HCl was added to lower the pH from 8.0 to 6.5, and CD spectra were recorded. tTF itself was used as the base line.

### Confocal microscopy

For confocal microscopy studies, HUVECs (5.0 × 10^3^) were seeded into confocal dishes (2 cm diameter) and maintained at 37°C and 5% CO_2_ in DMEM supplemented with 10% FBS. Twenty four hours later, the medium was removed, cells were washed twice with PBS and resuspended in PBS at a pH of 7.4 or 6.5 using phosphate buffers made by mixing appropriate proportions of Na_2_HPO_4_ and NaH_2_PO_4_. Alexa 594-labeled tTF-pHLIP (40 μg) was added to the cells. After 30 or 60 min, the cells were washed twice with PBS and Hoechst 33342 was added to stain cell nuclei. Images were analyzed with a FV1000-IX81 confocal laser scanning biological microscope (Olympus, Japan).

### Flow cytometry

Ten thousand HUVECs were analyzed in each sample. The cells were suspended in phosphate buffer at pH 6.5 or 7.4 in the presence or absence of Alexa 594-labeled tTF-pHLIP (40 μg) at 37°C for 1 h. After washing the cells to remove free tTF-pHLIP, they were resuspended in 500 μL PBS (pH 7.4) and analyzed by flow cytometry (Becton Dickinson, San Jose, CA).

### Tumor animal models

All animal experiments carried out in this study were approved by the Committee on the Ethics of Animal Experiments of the Health Science Center of Peking University (Beijing, China). Five week old BALB/c nude mice weighing 20–23 g were purchased from Beijing Vital River Laboratories. Human MDA-MB-231 breast cancer and B16-F10 melanoma cells (American Type Culture Collection) were grown as attached monolayer cultures in DMEM, and were afterwards dissociated in trypsin, washed and resuspended in PBS. MDA-MB-231 cells (2.0 × 10^6^) were mixed with 50 μL of Matrigel and injected into the mammary fat pads of the mice. For C57BL/6 or BALB/c nude models of melanoma, B16-F10 cells (1.0 × 10^6^) were injected subcutaneously into the right flank. Tumors were allowed to grow to a volume of approximately 100 to 200 mm^3^ (volume = [length × width^2^]/2; as measured with a vernier caliper). The mice were then used for *in vivo* imaging and therapeutic experiments.

### Multispectral optoacoustic tomography

A real-time multispectral optoacoustic tomography (MSOT) scanner (iThera Medical inVision128, Germany) was utilized to determine tumor blood perfusion by detecting oxyhemoglobin (HbO_2_) and hemoglobin (Hb) signals. MDA-MB-231 tumor-bearing mice were anesthetized with isoflurane and placed in a supine position in an animal holder. Cross-sectional multispectral optoacoustic image datasets of the tumors were acquired at different wavelengths in the near-infrared window (at 10 nm intervals), where the absorbance wavelengths of HbO_2_ and Hb differ widely and can thus be used to easily distinguish these two forms.

### Statistics

For the study of coagulation activity *in vitro*, data were compared using Student's *t* test. For section analysis in the therapeutic experiment, each treatment group consisted of 9 to 12 mice, from which one section per mouse was analyzed. For the tumor growth experiments, the differences in mean tumor volumes between groups were compared using one-way analysis of variance (ANOVA) with repeated measures followed by Tukey's HSD test. Cumulative survival curves between groups were compared using Kaplan-Meier analysis followed by the Log rank (Mantel-Cox) test. Statistics were calculated using SPSS 18.0. *P*-values less than .05 were considered statistically significant.

## SUPPLEMENTARY MATERIALS


