# Supplementary material for: pHLIP-mediated targeting of truncated tissue factor to tumor vessels causes vascular occlusion and impairs tumor growth
Source: Oncotarget. 2015 Jun 25;6(27):23523–32. doi: 10.18632/oncotarget.4395 (PMC4695134; doi:10.18632/oncotarget.4395)
Supplement: Supplementary file 1 [file oncotarget-06-23523-s001.pdf]

SUPPLEMENTARY MATERIALS

POPC liposome preparation

To prepare liposomes, 1-palmitoyl l-2-oleoyl-sn-glycero-3-phosphocholine (POPC; Avanti Polar Lipids, Inc.) dissolved in chloroform at a concentration of 25 mg/mL was desolvated on a rotary evaporator and dried under high vacuum for 20 min at room temperature. The phospholipid film was then rehydrated in 2 mL phosphate buffer (10 mM, pH 8.0), vortexed for 20 min, and sonicated for 30 seconds. The morphology of the resulting liposomes was confirmed using transmission electron microscopy (TEM, JEM-20 0CX, Jeol Ltd., Japan). The liposome size distribution was evaluated using a ZetaSizer Nano series Nano-ZS (Malvern Instruments Ltd., Malvern, UK).

Platelet activity analysis and determination of cerebral microthrombus

To determine the appropriate dose of tTF-pHLIP for *in vivo* applications, we evaluated the potential of different doses of tTF-pHLIP to activate circulating platelets. Platelet activation is thought to be critical for thrombosis, and is characterized by P-selectin expression on the platelet surface [1, 2]. Nude mice bearing MDA-MB-231 human breast tumor xenografts were injected intravenously with saline (negative control), thrombin (3.5 U/mouse; equivalent to approximately 145 U/kg body weight, positive control) or various doses of tTF-pHLIP (1, 10, 20, or 30 μg). At 30 min post-injection, whole blood was collected retro-orbitally into 3.8% trisodium citrate, and mixed with an equal volume of 2% paraformaldehyde for 30 min at room temperature. The blood was then centrifuged to obtain platelet-rich plasma (PRP). The PRP was incubated with a FITC-conjugated P-selectin-specific monoclonal antibody (BD Pharmingen, San Diego, CA) and then analyzed by FACSCalibur flow cytometry (Becton Dickinson, San Jose, CA). A dose of 30 μg/mouse induced slight platelet activation, so a relatively safe dose of 20 μg/mouse (equivalent to approximately 833 μg/kg) was used for *in vivo* experiments.

To verify the safety of the dose used, we examined cerebral microthrombus formation using a cranial window technique that has been shown not to cause artificial damage to normal blood flow [3]. Briefly, MDA-MB-231 tumor-bearing mice were injected with saline, tTF-pHLIP (20 μg/mouse) or thrombin (5.0 U/mouse) via a tail vein. At 30 min post-administration, the mice were anesthetized (50 mg/kg body weight of pentobarbital sodium) and placed in a prostrate position with their heads fixed between metal strips. A hole was drilled in the skull with a hand-held drill at the parietal cortex 4 mm posterior to the bregma and 2 mm lateral to the midline. A bolus of acridine red (12.5 mg/kg body weight) that specifically binds platelet aggregates and white blood cells was given to the mice via tail vein injection. With an observation window of ~0.5 × 0.5 cm^2, cerebral microthrombi within the vessels were imaged using a super sensitive CCD camera (USS-301, UNIQ Vision, Santa Clara, CA) attached to a microscope and examined using a color monitor (J2188A, TCL, Huizhou, China). Movies of blood flow were recorded with a DVD recorder (DVR41-R25, Malata, Xiamen, China). No microthrombi were found in the cerebral venous vessels of mice treated with tTF-pHLIP nor in saline-treated animals. In contrast, thrombin injection induced microthrombus formation as verified by platelet aggregates and slowed the flow of white blood cells. These data confirmed the safety of tTF-pHLIP at the dose of 20 μg/mouse.

REFERENCES

1. Michelson AD, Barnard MR, Krueger LA, Valeri CR, Furman MI. Circulating monocyte-platelet aggregates are a more sensitive marker of *in vivo* platelet activation than platelet surface P-selectin: studies in baboons, human coronary intervention, and human acute myocardial infarction. Circulation. 2001; 104:1533–1537.
2. Merten M, Thiagarajan P. P-selectin expression on platelets determines size and stability of platelet aggregates. Circulation. 2000; 102:1931–1936.
3. Tomita Y, Pinard E, Tran-Dinh A, Schiszler I, Kubis N, Tomita M, Seylaz J. Long-term, repeated measurements of mouse cortical microflow at the same region of interest with high spatial resolution. Brain Research. 2011; 1372:59–69.

SUPPLEMENTARY FIGURES AND TABLE

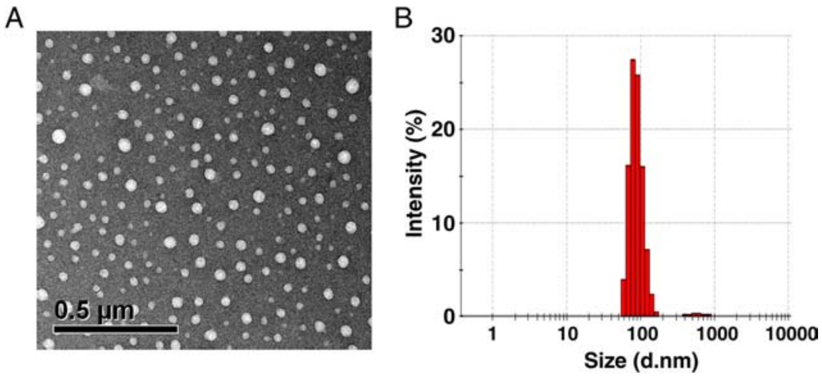

Supplementary Figure S1: Characterization of liposomes. A. Representative TEM images of POPC liposomes. **B.** Dynamic light scattering histogram showing the size distribution of the liposomes.

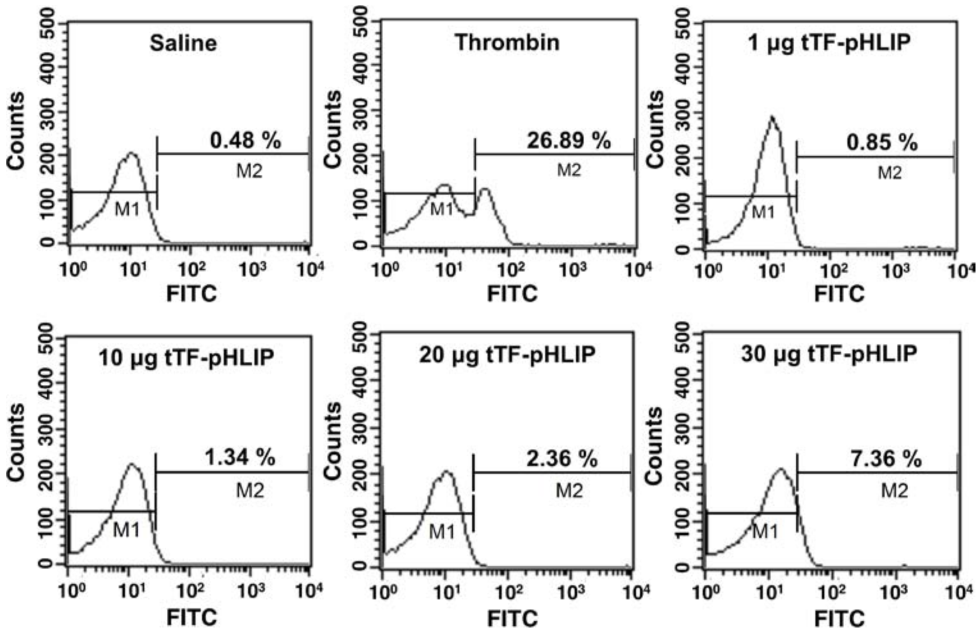

Supplementary Figure S2: Selection of the safe therapeutic dose of tTF-pHLIP by measuring platelet surface P-selectin. MDA-MB-231 tumor-bearing mice were injected intravenously with saline, thrombin (3.5 U/mouse) or tTF-pHLIP at 1, 10, 20, or 30 µg/mouse. Platelet surface P-selectin expression was measured and expressed as the percentage of platelets that are P-selectin-positive. The dose of 30 µg/mouse was found to induce slight platelet activation. The dose below this (20 µg/mouse) was therefore used for *in vivo* experiments. Data are representative of three separate experiments.

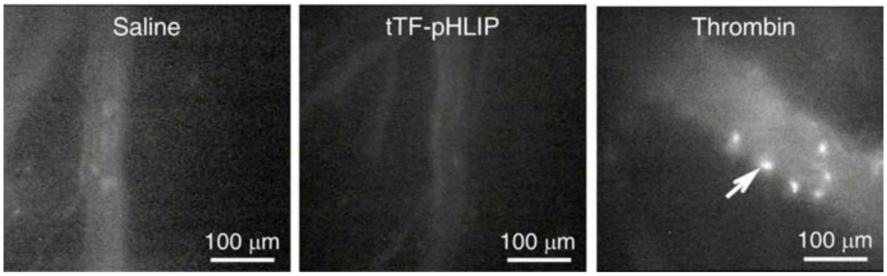

Supplementary Figure S3: Detection of cerebral microthrombi in mice. MDA-MB-231 tumor-bearing mice were injected with saline, tTF-pHLIP (20 μg/mouse) or thrombin (5.0 U/mouse) via a tail vein. Cerebral microthrombus formation was detected using the cranial window technique. Injection of tTF-pHLIP did not induce microthrombi in cerebral venous vessels, whereas injection with thrombin induced microthrombi as indicated by the arrows. Shown are representative images acquired by a DVD recorder.

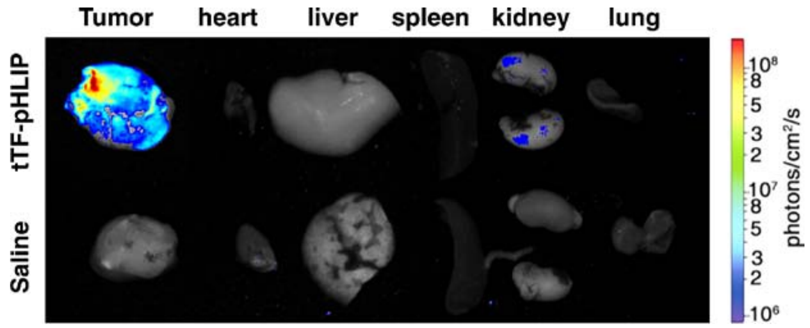

Supplementary Figure S4: Fluorescence imaging of representative organs after administration of tTF-pHLIP to tumor-bearing mice. Mice bearing MDA-MB-231 tumors were injected intravenously with saline or FAM-labeled tTF-pHLIP. After 8 hours, the mice were killed, and the tumors and other major organs were resected and imaged using an *in vivo* optical imaging system.

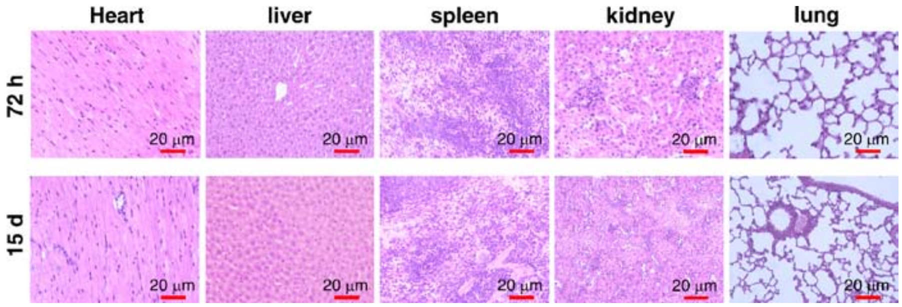

Supplementary Figure S5: H&E staining of normal tissue of the tTF-pHLIP-treated mice. Mice bearing MDA-MB-231 tumors were intravenously administered with a single dose (20 µg/mouse) or three doses of tTF-pHLIP at intervals of 6 d. After 72 hours or 15 days, the major organs were removed and stained with H&E for thrombosis detection. No thrombosis was observed in any tissue.

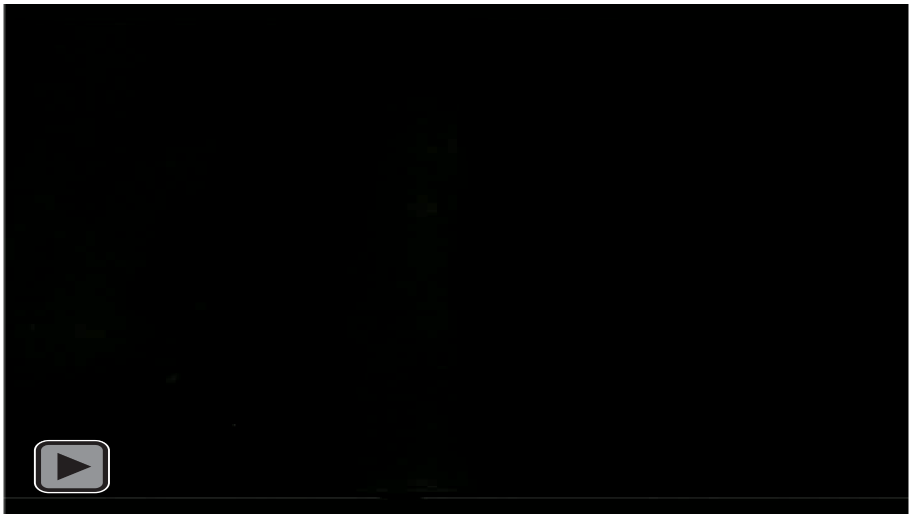

Supplementary Video S1: Movie showing blood flow in cerebral venous vessels of mice bearing MDA-MB-231 tumors 30 min post-injection with saline. No visible microthrombi were formed. Circulating objects indicate normal white blood cells

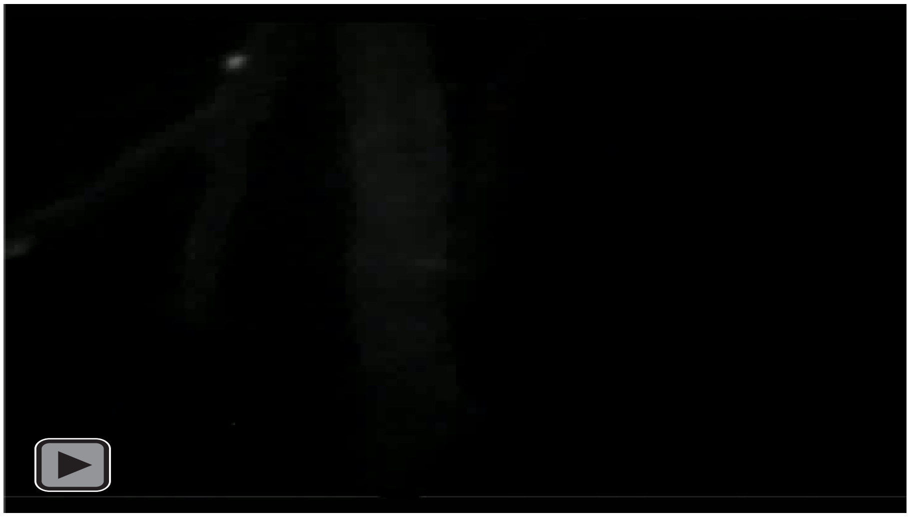

Supplementary Video S2: Movie showing blood flow in cerebral venous vessels of mice bearing MDA-MB-231 tumors 30 min post-injection with tTF-pHLIP (20 µg/mouse). No visible microthrombi were formed. Circulating objects indicate normal white blood cells.

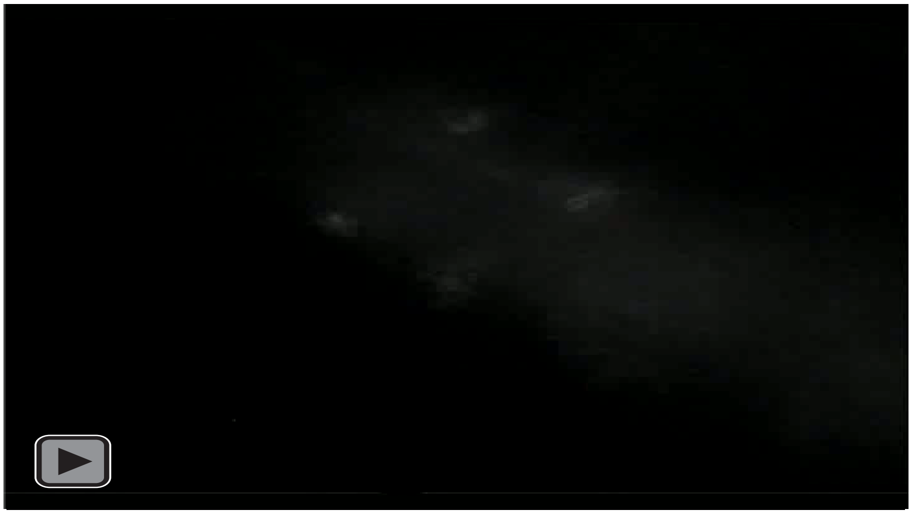

Supplementary Video S3: Movie showing blood flow in cerebral venous vessels of mice bearing MDA-MB-231 tumors 30 min post-injection with thrombin (5.0 U/mouse). Microthrombi occurred, as verified by the massive bright platelet aggregates along vessel walls. The circulating white blood cells also flow slowly.

Supplementary Table S1: Serum cytokine concentrations of non-tumor bearing mice after tTF-pHLIP treatment

Cytokine	Pre-injection	tTF-pHLIP (after single injection)	tTF-pHLIP (after multiple injections)
IL-6	10.9 ± 5.9	11.3 ± 4.1	13 ± 10
TNF-α	35.0 ± 8.7	35 ± 19	39 ± 16
IFN-α	0	0	0
IP-10	155 ± 54	157 ± 13	161 ± 20

Non-tumor-bearing C57BL/6 mice were injected intravenously with saline (negative control), a single dose of tTF-pHLIP, or three doses of tTF-pHLIP. Serum concentrations (pg/mL) of IL-6, IP-10, TNFα and IFNα were measured by ELISA (Thermo Scientific (IFNα), R&D (IL-6, IP-10, TNFα)) with 50 µL of serum, according to the manufacturers' protocols. All error values represent the standard deviation ($n = 5$).
